# YAP Activity is Not Associated with Survival of Uveal Melanoma Patients and Cell Lines

**DOI:** 10.1038/s41598-020-63391-z

**Published:** 2020-04-10

**Authors:** Yong Joon Kim, Sung Chul Lee, Sung Eun Kim, Seo Hee Kim, Sang Kyum Kim, Christopher Seungkyu Lee

**Affiliations:** 1 Department of Ophthalmology, The Institute of Vision Research, Severance Hospital, Yonsei University College of Medicine, Seoul, Republic of Korea; 2 Department of Pathology, Severance Hospital, Yonsei University College of Medicine, Seoul, Republic of Korea

**Keywords:** Eye cancer, Tumour biomarkers

## Abstract

Recent experimental studies have demonstrated an essential role for the Hippo-Yes-associated protein (YAP) pathway in *GNAQ/GNA11*-induced tumorigenesis in uveal melanoma (UM). However, the association between YAP activity and clinical outcomes remains elusive. We investigated possible associations between YAP activity and clinicopathological features including survival outcomes in patients with UM using The Cancer Genome Atlas (TCGA) cohort and our local cohort. We estimated YAP activity by mRNA expression levels, Gene Set Variation Analysis (GSVA) for the TCGA cohort, and immunohistochemical YAP staining for the local cohort. In the TCGA cohort, most clinicopathological features including tumor stage, mitotic counts, mutation of genes, and tumor sizes did not significantly differ between low and high YAP activity groups. In the local cohort, YAP nuclear-positive staining was observed in 30 (42%) of 72 patients with primary UM. UM-specific survival was not significantly different between tumors with low and high YAP activities. Unlike mesothelioma cells harboring a mutation of negative regulators of YAP, the survival of multiple UM cell lines was not significantly reduced by YAP/TAZ depletion. Our results suggest that the effect of YAP on development, growth, and invasion of UM in actual patients is less than previously demonstrated in experimental studies.

## Introduction

Uveal melanoma (UM) is the most common primary intraocular tumor of the adult population, comprising approximately 5% of all melanomas^1,2^. Primary UM responds favorably to radiotherapy, with a local recurrence rate of approximately 5%^1^. However, systemic metastasis occurs in up to 50% of patients with UM, even after enucleation, and there is currently no effective treatment option for UM metastasis^3^. Despite advances in chemotherapy including targeted therapy and immunotherapy, the therapeutic outcome of metastatic UM has not essentially improved^4,5^.

The introduction of next generation sequencing (NGS) has recently identified many genes, prognostic factors, and therapeutic targets associated with human cancers^6^. There have also been intense research efforts to develop effective drugs for UM using the results of NGS^7,8^. Most UM tumors harbor the *GNAQ* or *GNA11* mutations; these mutants have been shown to activate oncogenic pathways, including the mitogen-activated protein kinase (MAPK) and Hippo-Yes-associated protein (YAP) pathways^9–11^. Based on these studies, phase II/III clinical trials using the MEK1/2 inhibitor, selumetinib, have been conducted, but selumetinib monotherapy or combination therapy with dacarbazine has failed to increase the overall survival of patients with UM^12,13^.

Recent studies have reported that the Hippo-YAP pathway is important for cancer cell proliferation and maintenance of cancer stem cell-like properties in many human cancers^14,15^. In addition, hyperactive YAP is known to be important for metastasis or the acquisition of resistance to anticancer agents^16–19^. YAP mRNA expression levels and YAP-regulated molecular signatures have been shown to be prognostic factors in the survival of patients with pancreatic ductal adenocarcinoma and those with oral squamous cell carcinoma, respectively^20–22^. Immunohistochemical nuclear YAP staining has also been reported as a significant prognostic factor in adenocarcinomas of the ampulla of Vater^23^. *In vitro* studies and xenograft models have demonstrated an essential role for the Hippo-YAP pathway in GNAQ- and GNA11-induced tumorigenesis, and have suggested that YAP is a potential drug target for UM^10,11,24^. However, the results of basic and translational studies have not been validated in patients with UM and multiple UM cell lines. In this study, we investigated the association between YAP activity and clinicopathological characteristics in patients with UM using two clinical cohorts, The Cancer Genome Atlas (TCGA) cohort and a local cohort with resected tumor tissues. We also investigated the effect of YAP/Transcriptional coactivator with PDZ-binding motif (TAZ) depletion on survival of multiple UM cell lines.

## Results

### Study population

**Figure 1:**
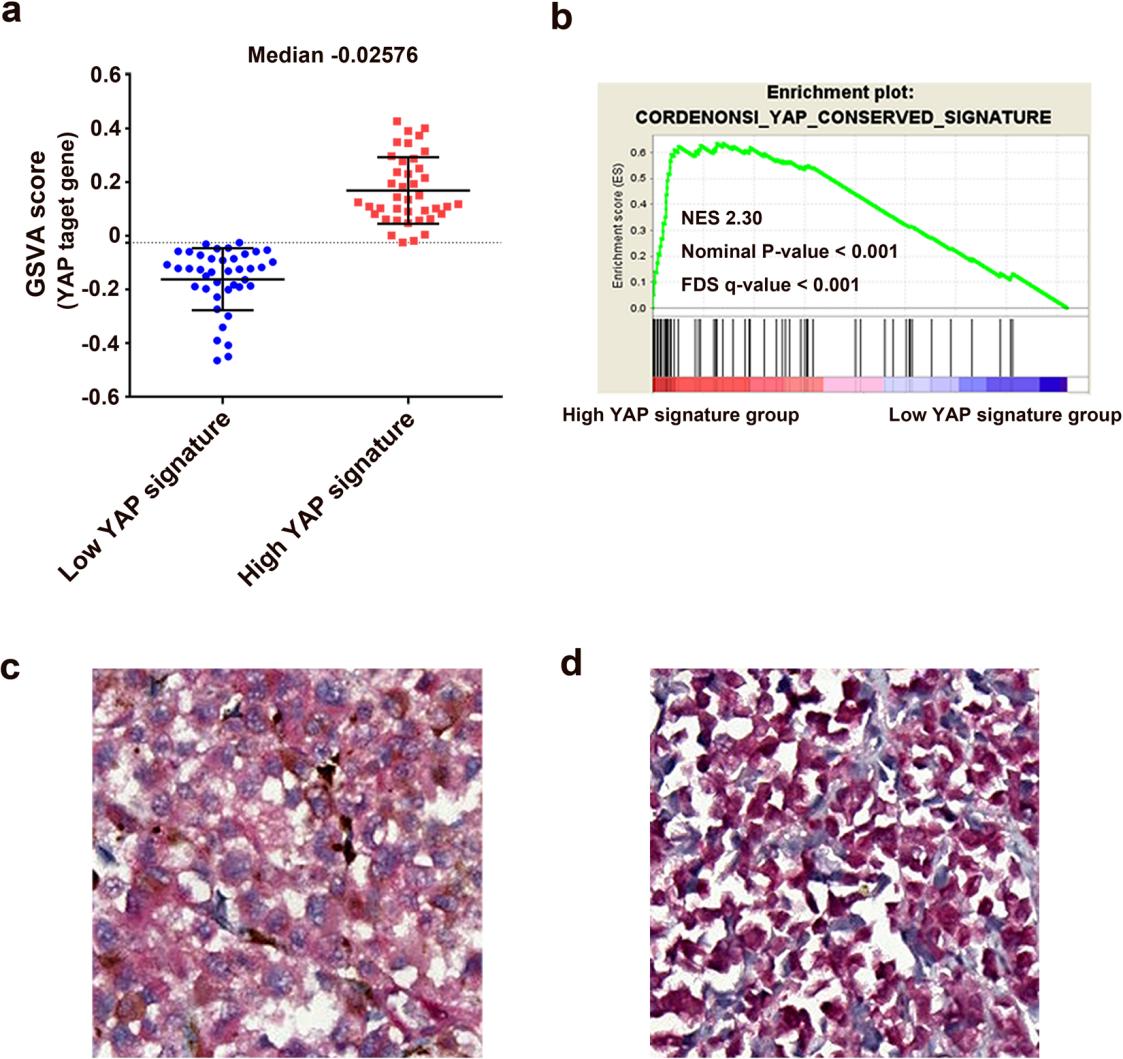
Estimated YAP activity based on Gene Set Variant Analysis of mRNA-seq data in the TCGA cohort, and immunohistochemical YAP staining in the local cohort. (**a**) Gene Set Variant Analysis Score for the YAP signature. (**b**) Gene Set Enrichment Analysis comparing low and high YAP signature groups. (**c**) Representative image of a YAP nuclear-negative tumor. (**d**) Representative image of a YAP nuclear-positive tumor.

For the TCGA cohort, all patients had UM with choroid or ciliary body involvement. Among them, two patients had UM involving the choroid, ciliary body, and iris. The mean RNA-seq by Expectation Maximization (RSEM)-normalized YAP mRNA levels were 1430.5 ± 362.4 and 2719.4 ± 700.8 for the low expression and high expression groups, respectively (*P* < 0.001). The mean Gene Set Variant Analysis (GSVA) scores for the YAP signature were 0.162 ± 0.115 and 0.168 ± 0.124 in the low YAP signature and high YAP signature groups, respectively (*P* < 0.001; Fig. 1a). When compared with the YAP-downregulated group, the YAP signature gene sets were significantly upregulated in the high YAP signature group (normalized enrichment score: 2.30; *P* < 0.001; Fig. 1b and Supplementary Fig. 1). The clinicopathological characteristics of the TCGA cohort are listed in Table 1.

**Table 1:** Clinicopathologic characteristics of patient groups in TCGA cohort.

	YAP mRNA expression level		P-value	YAP Signature Score (GSVA)		P-value
	Low YAP expression	High YAP expression		Low YAP signature	High YAP signature	
Number of Patients	40	40	—	40	40	—
Age, years, Mean (SD)	63.0 (15.0)	61.4 (13.1)	0.62	64.9 (13.2)	59.5 (14.4)	0.09
Male, No. (%)	23 (57.5%)	22 (55%)	0.99	22 (55%)	23 (57.5%)	0.99
Basal diameter (mm)*	16.3 ± 3.8	16.8 ± 3.8	0.72	15.9 ± 3.7	17.2 ± 3.9	0.13
Thickness (mm)*	10.3 ± 2.7	10.5 ± 2.9	0.7	10.3 ± 2.6	10.5 ± 3.0	0.79
AJCC stage (8th edition)						
Stage IIA, No. (%)	3 (7.5%)	1 (2.5%)	0.9	3 (7.5%)	1 (2.5%)	0.6
Stage IIB, No. (%)	17 (42.5%)	15 (37.5%)		17 (42.5%)	15 (37.5%)	
Stage IIIA, No. (%)	12 (30%)	15 (37.5%)		10 (25%)	17 (42.5%)	
Stage IIIB, No. (%)	5 (12.5%)	5 (12.5%)		6 (15%)	4 (10%)	
Stage IIIC, No. (%)	1 (2.5%)	2 (5%)		2 (5%)	1 (2.5%)	
Stage IV, No. (%)	2 (5%)	2 (5%)		2 (5%)	2 (5%)	
Dominant cell types						
Spindle, No. (%)	34 (85%)	23 (57.5%)	0.013	33 (82.5%)	24 (60%)	0.047
Epithelioid, No. (%)	6 (15%)	17 (42.5%)		7 (17.5%)	16 (40%)	
Pigmentation						
Minimal, No. (%)	14 (35%)	25 (62.5%)	0.025	19 (47.5%)	20 (50%)	0.99
Marked, No. (%)	26 (65%)	15 (37.5%)		21 (52.5%)	20 (50%)	
Mitotic count						
0-5/HPF, No. (%)	32 (80%)	31 (77.5%)	0.84	34 (85%)	29 (72.5%)	0.42
5-10/HPF, No. (%)	6 (15%)	5 (12.5%)		4 (10%)	7 (17.5%)	
>11/HPF, No. (%)	2 (5%)	4 (10%)		2 (5%)	4 (10%)	
GNAQ/11 mutation, No. (%)	37 (92.5%)	36 (90%)	0.99	38 (95%)	35 (87.5%)	0.43
BAP1 mutation, No. (%)	13 (32.5%)	22 (55%)	0.07	13 (32.5%)	22 (55%)	0.07
EIF1AX mutation, No. (%)	6 (15%)	4 (10%)	0.74	6 (15%)	4 (10%)	0.74
SF3B1 mutation, No. (%)	11 (27.5%)	7 (17.5%)	0.42	13 (32.5%)	5 (12.5%)	0.06

SD = standard deviation, No. = Number, AJCC = American Joint Committee on Cancer.

*Data are presented as mean ± standard deviation.

Regarding the local cohort, from January 2001 to December 2012, surgical resections of primary UM were performed in 74 patients; the resulting formaldehyde-fixed and paraffin-embedded tissues were available from 72 patients. Among these 72 patients, 61 and 11 were treated with enucleation and sclerouvectomy with brachytherapy, respectively. When immunohistochemical (IHC) staining for YAP was performed on these tissues, YAP nuclear-negative staining was observed in 42 patients, whereas YAP nuclear-positive staining was observed in 30 patients (Fig. 1c,d). The mean ages were 51.3 ± 14.6 and 53.6 ± 16.1 years in the YAP nuclear-negative and YAP nuclear-positive groups, respectively (*P* = 0.53). Twenty-four and 14 patients in the YAP nuclear-negative and YAP nuclear-positive groups, respectively, were male (*P* = 0.47).

### Largest basal diameter and thickness of tumors

**Figure 2:**
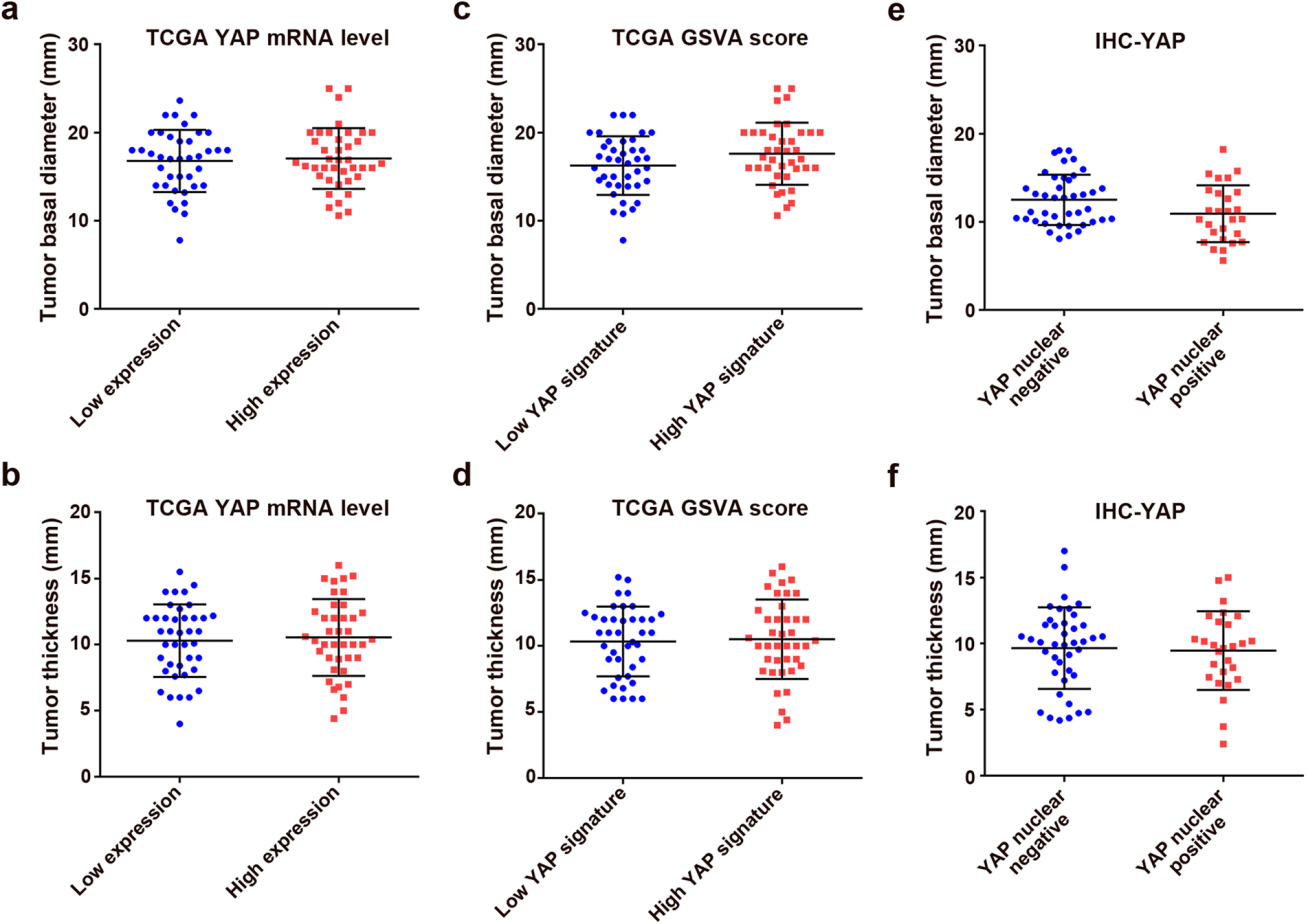
Comparison of tumor size according to YAP activity. (**a,b**) YAP mRNA low and high expression groups. (**c,d**) Low and high YAP signature groups. (**e,f**) YAP nuclear-negative and -positive groups. Error bars represent standard deviations.

In the TCGA cohort, based on the YAP mRNA expression levels, the largest basal diameters and thicknesses were not significantly different between the YAP mRNA low expression and high expression groups (*P* = 0.70 and *P* = 0.72, respectively; Fig. 2a,b and Table 1). When comparing the lower 25% and upper 25%, the largest tumor basal diameters and thicknesses also did not significantly differ (*P* = 0.36 and *P* = 0.74, respectively). When comparing the low YAP signature and high YAP signature groups, there were no significant differences in tumor basal diameter and thickness (*P* = 0.13 and *P* = 0.79, respectively; Fig. 2c,d and Table 1). No significant differences were observed in tumor diameter and thickness between the lower 25% and upper 25% of the GSVA scores for the YAP signature (*P* = 0.16 and *P* = 0.49, respectively).

In the TCGA cohort, the largest basal diameter and thickness were significantly correlated (*P* < 0.001; *R*^2^ = 0.205). However, the YAP mRNA levels were not associated with tumor diameter or thickness (*P* = 0.14 or *P* = 0.18, respectively; *R*^2^ = 0.028 or *R*^2^ = 0.023, respectively; Supplementary Fig. 2a,b). GSVA scores were also not correlated with tumor diameter or thickness (*P* = 0.06 or *P* = 0.42, respectively; *R*^2^ = 0.044 or *R*^2^ = 0.008, respectively; Supplementary Fig. 2c,d).

In the local cohort, when the YAP nuclear-negative and YAP nuclear-positive groups were compared, the largest basal diameter was significantly smaller in the YAP nuclear-positive group (12.5 ± 2.9 mm vs. 10.9 ± 3.2 mm; *P* = 0.038; Fig. 2e), whereas the tumor thicknesses were not significantly different between groups (9.5 ± 0.6 mm vs. 9.7 ± 0.5 mm; *P* = 0.81; Fig. 2f).

### UM-specific survival

**Figure 3:**
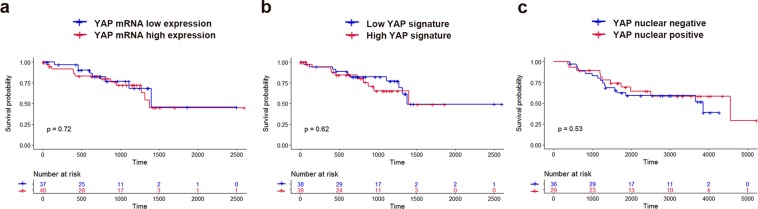
Kaplan–Meier curves showing uveal melanoma-specific survival for low and high YAP activities. (**a**) Comparison between low and high YAP mRNA expression groups (*P* = 0.72). (**b**) Comparison between low and high YAP signature groups (*P* = 0.62). (**c**) Comparison between YAP nuclear-negative and -positive groups (*P* = 0.53).

UM-specific survival was not significantly different between the low expression and high expression groups [hazard ratio (HR): 1.173; 95% confidential interval (CI): 0.483–2.850; *P* = 0.72; Fig. 3a]. UM-specific survival also did not differ between the low YAP signature and high YAP signature groups (HR: 1.272; 95% CI: 0.523–3.093; *P* = 0.62; Fig. 3b). Tumors that exhibited YAP nuclear-positive staining were not associated with poor UM-specific survival (HR: 0.786; 95% CI: 0.369–1.675; *P* = 0.53; Fig. 3c).

### Metastasis

In the local cohort, 19/42 (45%) and 19/30 (63%) patients developed metastases in the YAP nuclear-negative and YAP nuclear-positive groups, respectively. Liver metastasis was observed in 17 and 18 patients in the YAP nuclear-negative and -positive groups, respectively. Extrahepatic metastasis occurred in two patients in the YAP nuclear-negative group and one patient in the YAP nuclear-positive group. Details of the metastatic sites are listed in Supplementary Table 1.

### Comparison of IHC staining patterns between primary and metastatic UM tumors

There were 16 metastatic UM tumors (liver metastasis); nine (56%) showed a YAP nuclear-positive pattern using IHC staining. The YAP IHC staining patterns did not significantly differ between primary (n = 72) and metastatic (n = 16) UM tumors (*P* = 0.41).

### Effect of YAP knockdown on cell survival

**Figure 4:**
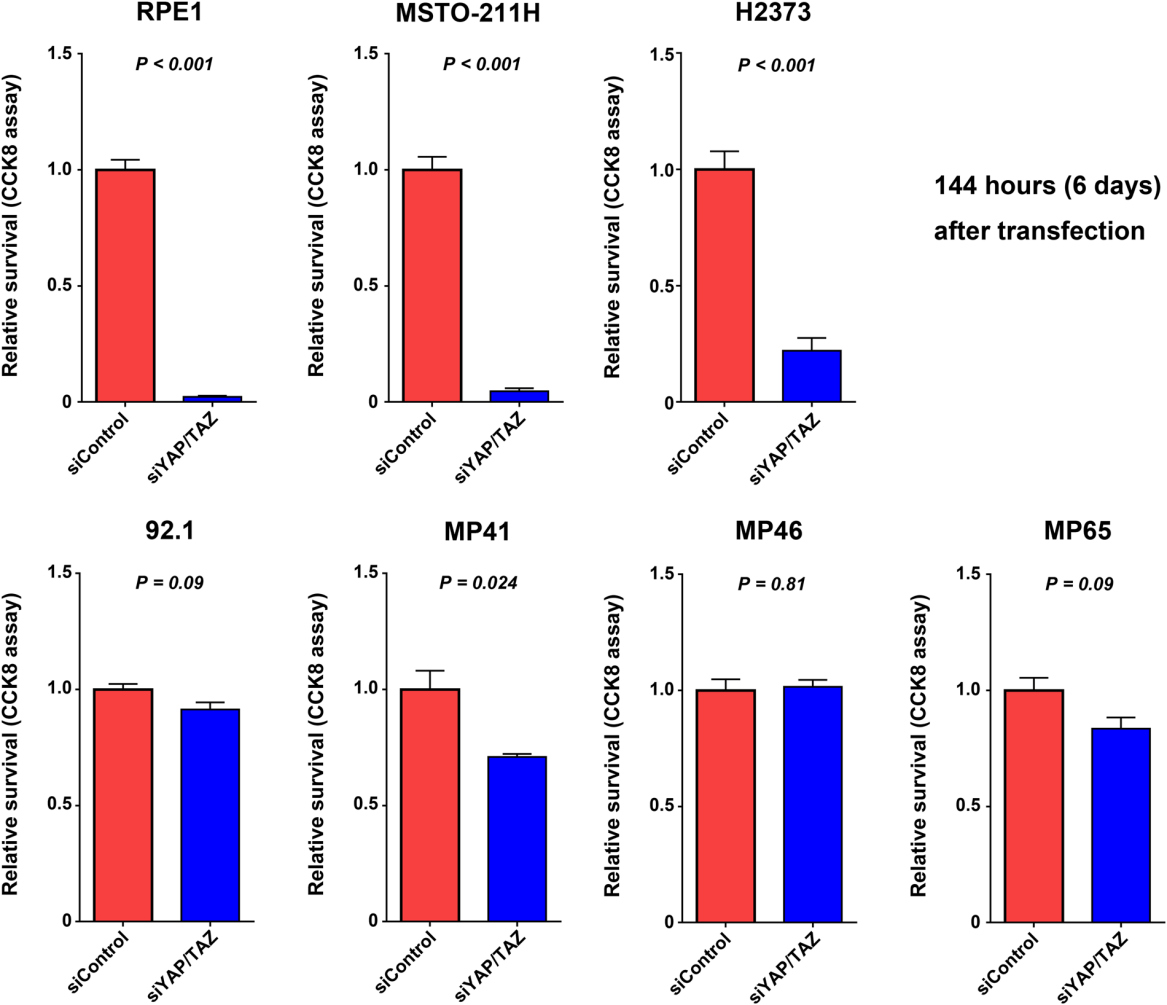
Effects of siRNA-mediated YAP knockdown on cellular proliferation in multiple uveal melanoma cell lines (92.1, MP41, MP46, and MP65) and other cell lines. Viable cells were quantified at 144 hours after siRNA transfection. Error bars represent standard error of the mean (n = 3 independent experiments).

The siRNA-mediated knockdown of YAP and its paralog, TAZ, significantly suppressed expression of YAP mRNA (Supplementary Fig. 3). In two mesothelioma cell lines [MSTO-211H (*LATS2* mutation) and H2373 (NF2 mutation)] and RPE1 cells, survival was significantly reduced at 72 hours after YAP/TAZ siRNA transfection (Supplementary Fig. 4). In these three cell lines, reduction in cell survival became evident over time. At 144 hours after siRNA transfection, relative survival proportions were 0.02 ± 0.01 (*P* < 0.001), 0.05 ± 0.02 (P < 0.001), and 0.28 ± 0.07 (*P* < 0.001) for the RPE1, MSTO-211H, and H2373 cells, respectively (Fig. 4).

YAP/TAZ knockdown exhibited smaller effects on survival in multiple UM cell lines (92.1, MP41, MP46, and MP65). Compared with RPE1, MSTO-211H, and H2373 cell lines, 92.1, MP41, MP46, and MP65 cell lines did not exhibit significant reduction in cell survival at 72 hours after siRNA transfection (Supplementary Fig. 4). Similar results were observed at 144 hours after transfection (Fig. 4). These results suggested that the effect of YAP on cell survival was much smaller in UM cell lines than in RPE1 cell line and mesothelioma cell lines.

## Discussion

Cancer genome sequencing efforts have identified frequently mutated genes in UM, including *GNAQ*, *GNA11*, *BAP1*, *SF3B1*, and *EIF1AX*^7,25^. Mutations of these genes are reportedly associated with the development or prognosis of UM^7,26,27^. Remarkably, *GNAQ* and *GNA11* are mutated in most UM tumors (~93%) with hotspot mutations (Q209P/L)^7^. Recent studies have highlighted the roles of *GNAQ* and *GNA11* mutations in UM development. *GNAQ* and *GNA11* mutations result in constitutive activation of oncogenic Gq/G11 subunits, leading to UM tumorigenesis by sequential activation of YAP^10,11,23^.

The Hippo-YAP signaling pathway is a key determinant of organ size, stem cell homeostasis, and cellular differentiation^14,16^. Two Hippo pathway transducers, YAP and its paralog TAZ, are transcriptional coactivators with nuclear-cytoplasmic distributions that are mainly controlled by Hippo signaling^28^. LATS1/2, MST1/2, and NF2 are the major upstream kinases of YAP, which cause YAP cytoplasmic retention and degradation by phosphorylating YAP^14^. Nuclear YAP (the active form of YAP) induces the expression of cell proliferative and anti-apoptotic genes, mainly by interacting with TEAD family transcription factors^14^. In experimental models using the 92.1 UM cell line, inhibition of YAP by short hairpin RNA or verteporfin suppressed the growth of UM^10,11^.

Previous studies have reported that high YAP activity is associated with poor prognoses in various cancers^14,22^. In this study, we investigated the association between YAP activity and clinicopathological characteristics using the TCGA cohort and a local cohort. Nucleocytoplasmic shuttling of YAP is rapidly changed by various cellular cues; YAP phosphorylated by Hippo kinases shows cytoplasmic retention, followed by rapid degradation by the proteasome^15^. Therefore, YAP mRNA levels may not reflect YAP activity. To overcome this limitation, we also estimated YAP activity by calculating the enrichment score for the YAP conserved signature genes by GSVA for each tumor sample. In the TCGA cohort, epithelioid cell type and marked pigmentation were associated with high YAP activity. However, the cancer stages, mitotic counts, and gene mutation profiles did not differ between groups. Consistently, the YAP mRNA expression levels and GSVA scores for YAP signatures were not significantly associated with tumor size and prognosis.

We further validated the clinical outcomes in the local cohort. With respect to YAP, because subcellular localization reflects activity, it is possible to estimate YAP activity by using IHC staining^28^. YAP nuclear staining, which indicated active YAP, was observed in only 30 (42%) of patients with UM; moreover, YAP IHC staining patterns were not significantly different between primary and metastatic tumors. Tumor size and prognosis were also not significantly different between the YAP nuclear-negative and YAP nuclear-positive groups.

Although YAP activities measured by several methods were not associated with the prognoses of patients with UM, YAP may be a therapeutic target for UM if suppression of YAP activity significantly affects the survival of UM cell lines. To confirm this possibility, we investigated whether siRNA-mediated YAP knockdown affected the survival of UM cell lines. YAP knockdown slightly reduced the survival of 92.1, MP41, MP46, and MP65 UM cell lines. However, these effects were much smaller than the effects observed for the two mesothelioma cell types, MSTO-211H (LATS2 mutation) and H2373 (NF2 mutation), which harbor mutations in negative regulators of YAP^29^. In the TCGA mesothelioma cohort, 35 (42%) of 83 patients had mutations in MST1/2, LAST1/2, or NF (negative upstream regulators of YAP). In another analysis, a high YAP signature was associated with poor prognoses in the TCGA mesothelioma cohort (HR: 2.675; 95% CI: 1.63–4.389; *P* < 0.001)^22^.

In conclusion, our results suggested that the effects of YAP on development, growth, and invasion of UM in affected patients were much less than the effects observed in experimental studies. However, YAP may contribute to the failure of anti-cancer therapies in patients with UM. It has been shown that YAP is important for acquisition of resistance to targeted therapy in tumors with *KRAS* and *BRAF* mutations, and that YAP can induce immune evasion by increasing the expression of PD-L1^16–19,30^. YAP activation by the *GNAQ* and *GNA11* mutations may explain the failure of MEK1/2 inhibitors or immunotherapy in patients with UM. Additional studies are therefore needed to determine the exact role of YAP in the treatment of UM.

## Methods

### The TCGA cohort and data acquisition

mRNA expression (RNA Seq V2, RSEM) data were obtained from the TCGA dataset for 80 patients with UM via Firebrowse (Broad Institute, Cambridge, MA, USA). RSEM is a tool for quantifying transcript abundances from RNA-seq data^31^. The TCGA provides RSEM-normalized data for RNA-seq; therefore, we used these data. Clinicopathological characteristics and tumor size data were also downloaded from Firebrowse. Updated follow-up survival data were downloaded using R package TCGA biolinks (http://bioconductor.org/packages/release/bioc/html/TCGAbiolinks.html).

### The local cohort

This study analyzed a consecutive cohort of patients with UM who underwent surgical resection of primary tumors (enucleation or sclerouvectomy, 72 patients) or metastatic tumors (liver metastasis, 16 patients) at Severance Hospital (Yonsei University College of Medicine, Seoul, Republic of Korea) between January 2001 and December 2012. The medical records of patients who met the following inclusion criteria were retrospectively reviewed: (1) pathological confirmation; (2) presence of formaldehyde-fixed, paraffin embedded tissue blocks with adequate remaining tissue for additional immunohistochemical IHC staining of slides; and (3) available clinical follow-up data. This research adhered to the tenets of the Declaration of Helsinki. The Institutional Review Board/Ethics Committee approved this retrospective study (Severance Hospital, Yonsei University Health System, IRB no. 4-2016-0300), and informed consent for the surgical procedure was obtained from all participants before surgery.

### Gene set enrichment analysis (GSEA) and GSVA analysis

GSEA is a popular framework for condensing information from gene expression profiles into a pathway or signature summary^32^. GSVA is a method that estimates variation of pathway activity over a sample population^33^. We used the GSEA and GSVA methods to estimate the YAP activity from RNA-seq data. GSVA was performed using the R package GSVA. A list of genes for the YAP signature was determined using MSigDB (CORDENONSI_YAP_CONSERVED_SIGNATURE, Broad Institute)^34^. The normalized count data calculated by expectation maximization analysis were incorporated as a matrix in R^31^. The enrichment score was then calculated by GSVA for each tumor sample in the matrix data using the following arguments: kcdf = “Poisson”, min.sz = 5, max_sz = 500, mx.diff = TRUE. GSEA was performed using the C6 MSigDB gene set database to test whether the YAP signature was enriched in the high YAP signature group, compared with the low YAP signature group^32^. For detailed information, see: http://software.broadinstitute.org/gsea/index.jsp and Supplementary Table 2.

### IHC staining for YAP

A representative area of each tumor on the formalin-fixed, paraffin-embedded block was punched out and a 5-mm tissue core was placed into a recipient block. Two tissue cores were extracted to minimize extraction bias. To generate a tissue microarray, each tissue core was assigned. Tissue microarray blocks were used to obtain 5-μm-thick sections. IHC was performed with an anti-YAP antibody (dilution: 1:200; sc-101199; Santa Cruz Biotechnology, Dallas, TX, USA) in an automated immunohistochemical staining instrument (Ventana BenchMark XT; Ventana Medical System, Oro Valley, AZ, USA), in accordance with the manufacturer’s instructions. An Ultraview Universal Alkaline Phosphatase Red Detection Kit (Roche Diagnostics, Risch-Rotkreuz, Switzerland) was used for detection. IHC staining of all slides was interpreted by an expert melanoma pathologist (Sang Kyum Kim). The expression of nuclear YAP was semi-quantitatively evaluated by calculating the total immunostaining score (TIS), which is the product of the intensity score (IS), and proportion score (PS). IS represents the estimated staining intensity compared with that of control cells (0, no staining; 1, weak; 2, moderate; 3, strong); PS describes the estimated area of positively stained tumor cells (0, none; 1, <10%; 2, 10–50%; 3, 51–80%; 4, >80%). The TIS (IS × PS) ranges from 0 to 12 with only 10 possible values (0, 1, 2, 3, 4, 6, 7, 8, 9, and 12). The TIS was used to define the YAP nuclear-negative group (TIS 0–3) and YAP nuclear-positive group (TIS 4–12).

### Study groups

For the TCGA cohort, patients were classified according to YAP mRNA levels and GSVA scores for the YAP signature as follows: (1) low expression (lower 50%, n = 40) vs. high expression (upper 50%, n = 40) for the YAP mRNA levels; and (2) low YAP signature (lower 50%, n = 40) vs. high YAP signature (upper 50%, n = 40) for the GSVA scores. Based on the IHC results, patients were divided into two groups: YAP nuclear-negative and YAP nuclear-positive.

### Outcome measures

The main outcome measure was the difference in UM-specific survival between the two groups according to YAP activity. The other clinicopathological features included patient age, sex, American Joint Committee on Cancer (AJCC; 8th edition) stage, dominant cell type, pigmentation, mitotic count, largest tumor basal diameter, tumor thickness, presence or absence of metastasis, and metastatic sites.

### *In vitro* cell culture and siRNA-mediated YAP knockdown

UM cells (MP41 MP46, and MP65) and RPE1 cells were acquired from the American Type Culture Collection (ATCC, Manassas, VA, USA)^33^. MSTO-211H, H2373 and 92.1 cells were kind gifts from Joon Kim (KAIST, Daejeon, Republic of Korea)^35^. RPE1 cells were cultured in DMEM/F12 (Welgene, Kyungsan-si, Republic of Korea); other cells were cultured in RPMI-1640 (Welgene) supplemented with 20% fetal bovine serum (Welgene) and 1% penicillin/streptomycin (Welgene). siRNA transfection was performed with Lipofectamine RNAiMAX (Invitrogen, Carlsbad, CA, USA), in accordance with the manufacturer’s reverse transfection instructions. Allstars Negative Control siRNA (Qiagen, Hilden, Germany) was used as a control siRNA. Published and validated sequences of siRNA for YAP/TAZ were used as follows: YAP1: 5′-GACAUCUUCUGGUCAGAGAdTdT-3′ and TAZ: 5′-ACGUUGACUUAGGAACUUUdTdT-3′^28^. Each siRNA was used at a final concentration of 10 nM.

### Quantitative RT-PCR

Total RNA was extracted from cells using an RNeasy kit (Qiagen). A total of 1 μg of extracted RNA was annealed with oligo dT primer (Roche Life Science, Basel, Switzerland), and reverse-transcribed to cDNA using M-MLV reverse transcriptase (Promega, Madison, WI, USA) in the presence of RNase Inhibitor (RNasin Plus; Promega). cDNA was mixed with primers and iQ SYBR Green Supermix (Bio-Rad, Hercules, CA, USA), and mRNA expression levels were measured by real-time qRT-PCR using a CFX96 system (Bio-Rad). qRT-PCR primers used in this study were: glyceraldehyde 3-phosphate dehydrogenase (GAPDH) forward: 5′-CAACGGATTTGGTCGTATTG-3′, GAPDH reverse: 5′-GCAACAATATCCACTTTACCAGAGTTAA-3′, YAP forward: 5′-AATTTGCCCAGTTATACCTCAGTG-3′, and YAP reverse: 5′-CACATCAAGGCTATGATTCAAACTC-3′.

### Cell survival analysis

Cells were seeded on Black/Clear flat-bottomed 96-well plates (Falcon, Corning, NY, USA) at 3,500–5,000 cells per well for reverse transfection of siRNA. Cells were then incubated for 72 and 144 hours; viable cells were quantified using Cell Counting Kit-8 (Dojindo Molecular Technologies, Rockville, MD, USA).

### Statistical analysis

Statistical analyses were performed using SPSS statistical software for Windows, version 25.0 (IBM Corp., Armonk, NY, USA); Prism software, version 6 (GraphPad Software, La Jolla, CA, USA); or R software version 3.5.2 (R Foundation for Statistical Computing, Vienna, Austria). The chi-squared test and Fisher’s exact test were used to compare two groups. Pearson’s correlation coefficient was used to measure statistical relationships. The Kaplan–Meier method and log-rank test were used for comparisons of patient survival. All tests were two-tailed, and P < 0.05 was considered to be statistically significant.

## Supplementary information

Supplementary Information
