# Supplementary material for: YAP Activity is Not Associated with Survival of Uveal Melanoma Patients and Cell Lines
Source: Sci Rep. 2020 Apr 10;10:6209. doi: 10.1038/s41598-020-63391-z (PMC7148330; doi:10.1038/s41598-020-63391-z)
Supplement: Supplementary file 1 — Supplementary Information [file 41598_2020_63391_MOESM1_ESM.pdf]

Supplementary Materials for:

YAP Activity Is Not Associated with Survival of Uveal Melanoma Patients and Cell Lines

Yong Joon Kim[1], Sung Chul Lee[1] , Sung Eun Kim[1] , Seo Hee Kim[1] , Sang Kyum Kim[2,*] , Christopher Seungkyu Lee[1,*]

[1]Department of Ophthalmology, The Institute of Vision Research, Severance Hospital, Yonsei University College of Medicine, Seoul, Republic of Korea.

[2]Department of Pathology, Severance Hospital, Yonsei University College of Medicine, Seoul, Republic of Korea.

*Co-corresponding authors

Supplementary Materials

Supplementary Figure 1. Heat map of genes with core enrichment using Gene Set Enrichment Analysis in high and low YAP signature groups.

Supplementary Figure 2. Correlation between YAP activity and tumor size.

Supplementary Figure 3. qRT-PCR analyses of YAP mRNA expression level after the treatment of control siRNA and YAP/TAZ siRNA for 48 h.

Supplementary Figure 4. Effects of siRNA-mediated YAP knockdown on cellular proliferation in multiple uveal melanoma cell lines (92.1, MP41, MP46, and MP65) and other cell lines.

Supplementary Table 1. List of metastatic sites in the YAP nuclear positive and negative groups.

Supplementary Table 2. List of genes used for the YAP signature analysis (from MsigDB)

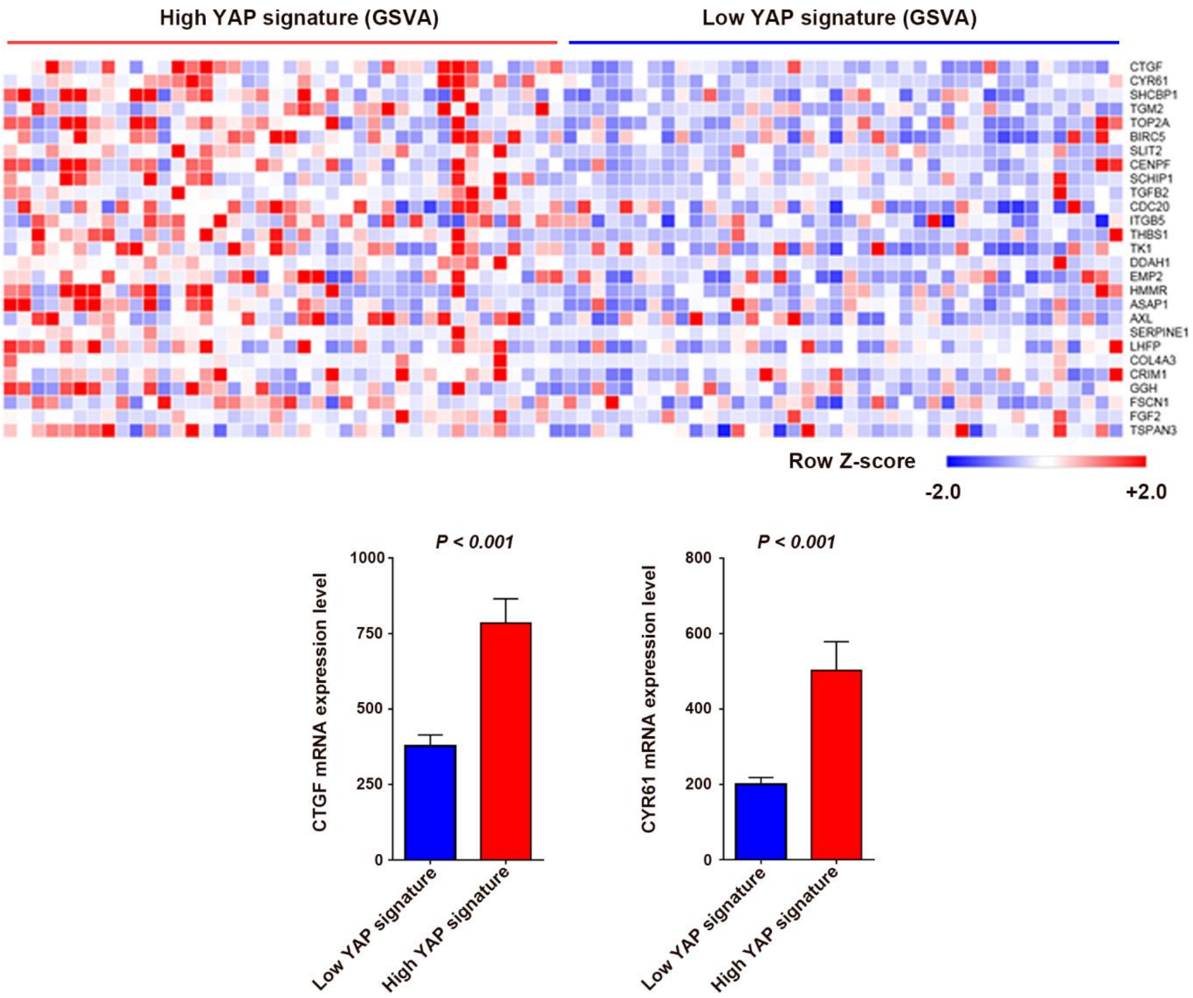

Supplementary Figure 1. Heat map of genes with core enrichment using Gene Set Enrichment Analysis in high and low YAP signature groups. Gene expression level of two representative YAP target gene, CTGF and CYR61 in high and low YAP signature groups, are also shown. Error bars represent SEM.

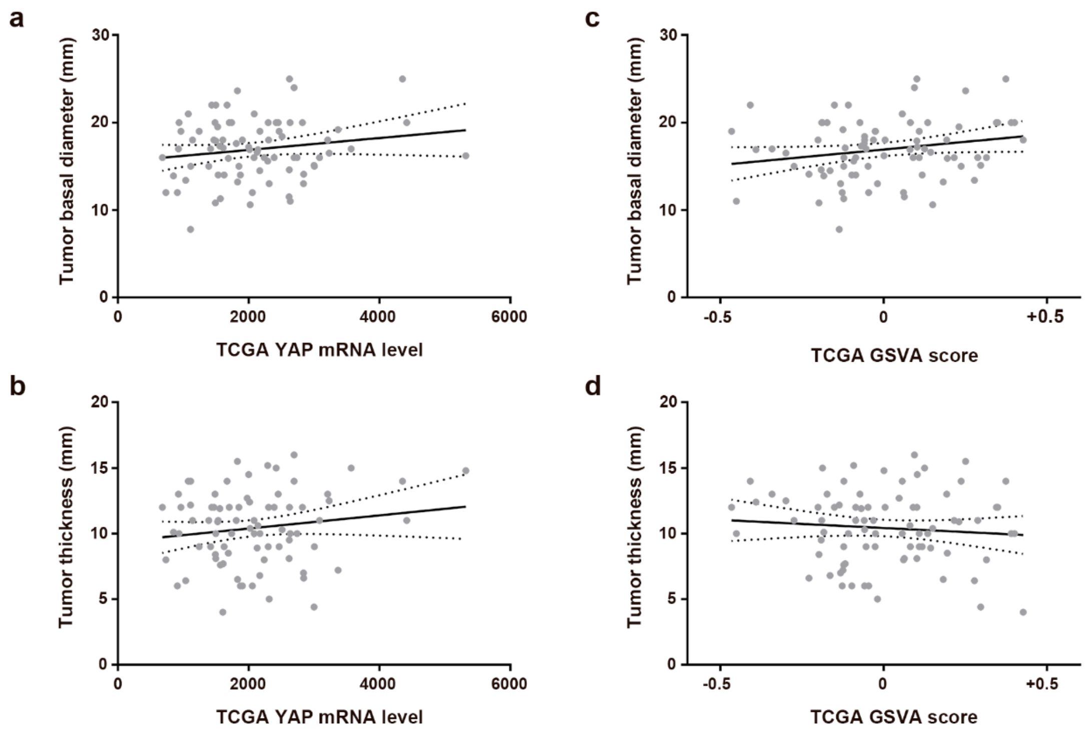

Supplementary Figure 2. Correlation between YAP activity and tumor size. (a and b) YAP mRNA expression levels and tumor sizes. (c and d) The Gene Set Variant Enrichment scores for YAP signature and tumor size.

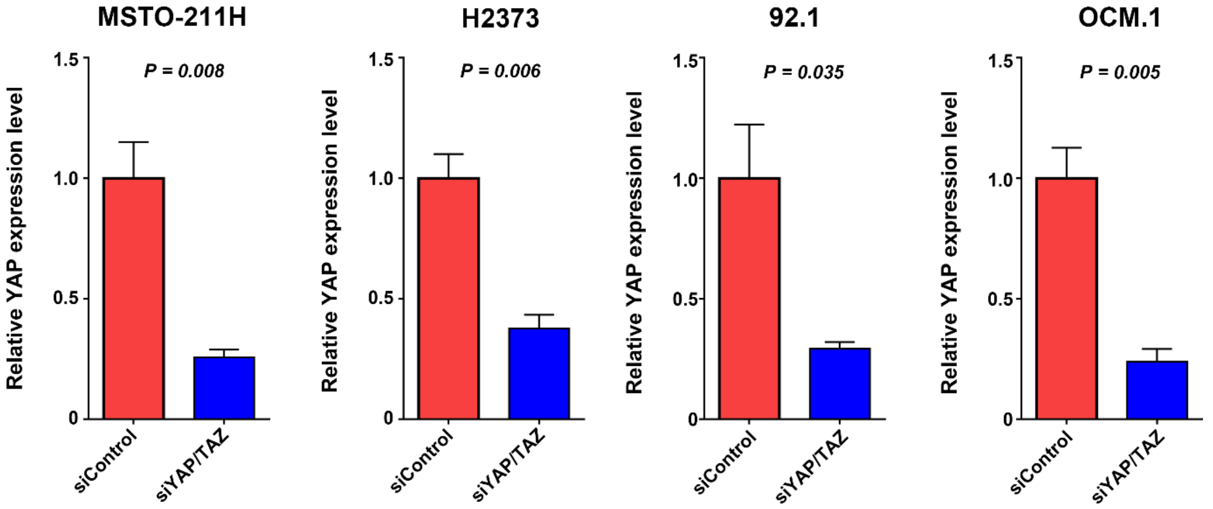

Supplementary Figure 3. qRT-PCR analyses of YAP mRNA expression level after the treatment of control siRNA and YAP/TAZ siRNA for 48 h. Error bars represent SEM (n = 3 independent experiments)

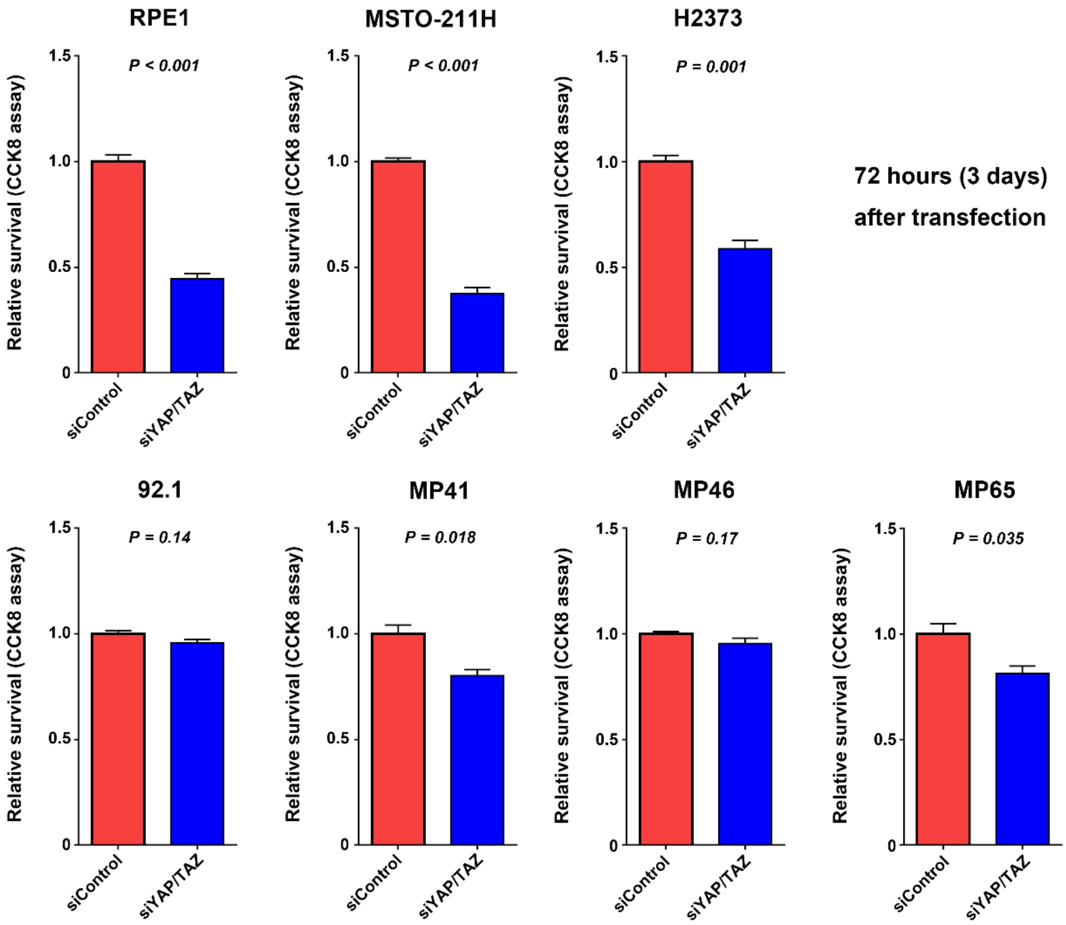

Supplementary Figure 4. Effects of siRNA-mediated YAP knockdown on cellular proliferation in multiple uveal melanoma cell lines (92.1, MP41, MP46, and MP65) and other cell lines. Viable cells were quantified at 72 hours after siRNA transfection. Error bars represent standard error of the mean (n = 3 independent experiments).

Supplementary Table 1. List of metastatic sites in the YAP nuclear negative and positive groups.

YAP nuclear negative				YAP nuclear positive			
No.	Age (y)	Sex	Metastasis site	No.	Age (y)	Sex	Metastasis site
1	66	F	Liver	20	30	F	Liver, Lung
2	66	M	Liver	21	43	F	Liver
3	31	F	Lung	22	48	M	Liver
4	61	M	Liver, Lung, Pleura, Bone	23	33	M	Liver
5	54	F	Liver	24	45	F	Liver
6	48	F	Liver, Lung, Abdominal wall	25	25	F	Liver
7	55	F	Bladder	26	54	F	Liver, Kidney
8	42	F	Liver	27	70	F	Liver
9	45	M	Liver	28	52	F	Liver, lung, Bone, LN
10	63	M	Liver	29	57	M	Liver
11	49	M	Liver, Adrenal gland	30	78	M	Liver, Bone
12	26	M	Liver	31	19	M	Liver, Bone
13	55	F	Liver, Spleen, Spine	32	52	F	Liver, Lung, Pancreas, Bone, Abdominal wall
14	51	M	Liver, Bone	33	58	M	Brain, Bone, Stomach
15	37	F	Liver, Bone	34	73	M	Liver
16	49	M	Liver, Lung, Bone	35	55	M	Liver, Lung, Bone
17	50	M	Liver	36	57	F	Liver, Bone
18	67	M	Liver	37	51	M	Liver, Kidney, LN
19	57	M	Liver	38	46	M	Liver

y = years, M = male, F = female

Supplementary Table 2. List of genes used for the YAP signature analysis (from MsigDB).

NCBI (Entrez) Gene ID	Gene symbol	NCBI (Entrez) Gene ID	Gene symbol
10397	NDRG1	2013	EMP2
3925	STMN1	27063	ANKRD1
10099	TSPAN3	3161	HMMR
7052	TGM2	11167	FSTL1
1843	DUSP1	1854	DUT
7057	THBS1	23576	DDAH1
3693	ITGB5	1490	CTGF(= CCN2)
1601	DAB2	1063	CENPF
3491	CYR61 (= CCN1)	4616	GADD45B
7153	TOP2A	9353	SLIT2
26227	PHGDH	7042	TGFB2
6624	FSCN1	5376	PMP22
4082	MARCKS	10395	DLC1
6446	SGK1	2744	GLS
3074	HEXB	80114	BICC1
332	BIRC5	2316	FLNA
2621	GAS6	7139	TNNT2
7083	TK1	1285	COL4A3
51232	CRIM1	29969	MDFIC
5054	SERPINE1	10186	LHFPL6
558	AXL	8436	CAVIN2
3689	ITGB2	7145	TNS1
991	CDC20	64236	PDLIM2
51421	AMOTL2	79801	SHCBP1
2119	ETV5	63898	SH2D4A
8836	GGH	1894	ECT2
29970	SCHIP1	50807	ASAP1
1031	CDKN2C	3268	AGFG2
2247	FGF2		
